# Can a plant-based diet help mitigate Covid-19?

**DOI:** 10.1038/s41430-022-01082-w

**Published:** 2022-01-21

**Authors:** Hana Kahleova, Neal D. Barnard

**Affiliations:** 1 Physicians Committee for Responsible Medicine, 5100 Wisconsin Ave, N.W. Ste.400, Washington, DC 20016 USA; 2 George Washington University School of Medicine and Health Sciences, Washington, DC USA

**Keywords:** Health care, Nutrition

As Covid-19 resurges, propelled by the emergence of the omicron variant, and by the reduced effectiveness of the Covid vaccines over time [1], many people are asking what can be done to prevent “breakthrough” infections in vaccinated people and what can turn the tide on the pandemic overall. Recent studies show what may be a surprising answer.

The effectiveness of vaccination, as measured by antibody titres, may be significantly reduced by the presence of chronic conditions, particularly central obesity, hypertension, dyslipidemia, and smoking [2]. Covid-19 deaths are considerably more common in the context of advanced age or chronic diseases, such as excess body weight, coronary heart disease, hypertension, type 2 diabetes, and chronic lung disease. In turn, these conditions are strongly linked to diet and lifestyle choices that can be changed. Indeed, those people who have made lifestyle changes have demonstrable protection against the virus. Analyzing data from 592,571 participants, Merino et al. showed that a dietary pattern characterized by healthy plant-based foods was associated with a 9% lower risk of Covid-19 infection and a 41% lower risk of severe Covid-19 [3]. The dietary data from 568 Covid-19 cases and 2316 controls from healthcare workers from six countries (France, Germany, Italy, Spain, UK, USA) with substantial exposure to Covid-19 patients have demonstrated that those following a plant-based diet had a 73% lower risk of moderate-to-severe Covid-19 [4].

Needless to say, plant-based diets were already beneficial for human health before the emergence of SARS-CoV-2. The Global Burden of Disease Study showed that low fruit, vegetable, and whole grain consumption, and, in contrast, high red and processed meat consumption are currently among the main global risks [5]. Proper nutrition may help prevent almost half of cardio-metabolic deaths in the US [5]. Specifically, a plant-based diet seems to decrease all-cause mortality and risk of obesity, type 2 diabetes, and coronary heart disease, partly by effectively improving nutrient intake [6], and thereby addressing the underlying health conditions that put our population at such a great risk of severe Covid mortality and morbidity.

In the context of Covid-19, we can draw a lesson from the “Blue Zones,” areas of particularly high longevity [7]. People in Okinawa, for example, consume a predominantly plant-based diet rich in phytochemicals and antioxidants, with over half of their daily caloric intake coming from sweet potatoes. They also consume abundant green leafy vegetables and soy products, with minimal fat (about 6% of the total energy intake) [8].

In addition to their high life expectancy and low mortality from cardiovascular disease and certain types of cancers, Okinawans have enjoyed a remarkable resistance to Covid-19 mortality. As of June 16, 2021, the Covid-19 mortality in Okinawa, Japan, was 0.08% (163 deaths out of 19,782 cases), which is one-sixteenth that of Tokyo (mortality rate 1.3%; 2183 deaths out of 167,416 cases) [9]. Okinawa is roughly the size of Tokyo, and vaccination rates in July were low in both. Lower population density in Okinawa could partly explain the lower Covid-19 toll. But it is likely that the remarkably low prevalence of chronic diseases in Okinawa has been a major factor.

In order to tackle the challenges of the Covid-19 delta variant surge, we must address the major lifestyle risk factors, such as smoking, lack of physical activity, and, most of all, poor diet. Adopting a healthful plant-based diet and lifestyle is a powerful tool which may delay the aging process, decrease age-associated co-morbidities, and decrease the risk of severe Covid-19 and mortality. It represents the most cost-effective approach and should be largely promoted and incorporated in everyday practice. This is a booster that is needed at this unprecedented time and that may actually work to mitigate Covid-19.
